# Intravenous whole blood transfusion results in faster recovery of vascular integrity and increased survival in experimental cerebral malaria

**DOI:** 10.1590/0074-02760220184

**Published:** 2023-01-20

**Authors:** Saba Gul, Hans C Ackerman, Cláudio Tadeu Daniel-Ribeiro, Leonardo JM Carvalho

**Affiliations:** 1 Fundação Oswaldo Cruz-Fiocruz, Instituto Oswaldo Cruz, Laboratório de Pesquisa em Malária, Rio de Janeiro, RJ, Brasil; 2 National Institutes of Health, National Institute of Allergy and Infectious Diseases, Laboratory of Malaria and Vector Research, Rockville, MD, USA

**Keywords:** experimental cerebral malaria, blood transfusion, artemether, adjunctive therapy, endothelial dysfunction

## Abstract

**BACKGROUND:**

Cerebral malaria is a lethal complication of *Plasmodium falciparum* infections in need of better therapies. Previous work in murine experimental cerebral malaria (ECM) indicated that the combination of artemether plus intraperitoneal whole blood improved vascular integrity and increased survival compared to artemether alone. However, the effects of blood or plasma transfusion administered via the intravenous route have not previously been evaluated in ECM.

**OBJECTIVES:**

To evaluate the effects of intravenous whole blood compared to intravenous plasma on hematological parameters, vascular integrity, and survival in artemether-treated ECM.

**METHODS:**

Mice with late-stage ECM received artemether alone or in combination with whole blood or plasma administered via the jugular vein. The outcome measures were hematocrit and platelets; plasma angiopoietin 1, angiopoietin 2, and haptoglobin; blood-brain barrier permeability; and survival.

**FINDINGS:**

Survival increased from 54% with artemether alone to 90% with the combination of artemether and intravenous whole blood. Intravenous plasma lowered survival to 18%. Intravenous transfusion provided fast and pronounced recoveries of hematocrit, platelets, angiopoietins levels and blood brain barrier integrity.

**MAIN CONCLUSIONS:**

The outcome of artemether-treated ECM was improved by intravenous whole blood but worsened by intravenous plasma. Compared to prior studies of transfusion via the intraperitoneal route, intravenous administration was more efficacious.

Despite advances in medical science and global technical strategies for malaria control, there were an estimated 241 million malaria cases with 627,000 deaths in 2020.^1^ Cerebral malaria (CM) is prominently involved in a majority of malaria related deaths, and frequently results in long-term cognitive deficits in survivors.^2^ ^,^ ^3^ Most of deaths occur in the first 24-48 hours of hospital admission, affording only a narrow time window in which to administer treatments to reduce the risk of death.^4^ ^,^ ^5^ Adjuvant therapies are urgently needed to increase survival and improve neurological outcomes from cerebral malaria.

Cerebral malaria is a multifactorial disorder accompanied by pathological hematological and vascular changes. These including anemia, altered rheologic properties of red cells, and thrombocytopenia, as well as alterations in the levels of angiopoietins, haptoglobin, L-arginine, and proinflammatory cytokines.^6^ ^-^ ^12^ Recently, a multicenter study in Africa showed that among children hospitalized with severe *Plasmodium falciparum* malaria complicated by impaired consciousness or severe acidosis, whole blood transfusion was associated with increased survival.^13^ ^,^ ^14^ A similar association was reported in artemether-treated murine experimental cerebral malaria (ECM) in which intraperitoneal whole blood administered in the symptomatic late stages of the infection restored hematocrit, preserved vascular integrity, and increased survival.^15^ The beneficial effects of intraperitoneal whole blood transfusion in ECM go hand-in-hand with prior evidence indicating that adjunctive therapies that restore vascular function are beneficial as adjunctive therapy in ECM.^16^ ^,^ ^17^ ^,^ ^18^ ^,^ ^19^

The prior study administered blood via the peritoneum because this site of administration was more feasible than injection into the tail vein, through which blood flow is restricted by venoconstriction and cytoadherence.^16^ ^,^ ^18^ ^-^ ^23^ The downsides of peritoneal blood administration in this experimental model is a diminution of effect and delay in response to transfusion^24^ and dissimilarity with blood transfusion in the human clinical setting which is administered intravenously. Therefore, in the present study we administered whole blood or plasma via the right jugular vein as adjunctive therapy to artemether in mice with artemether-treated late-stage ECM. The effect of plasma transfusion was also assessed. The results show that adjunctive treatment with whole blood via the intravenous route achieved survival rates of 90%, compared to 76% via the intraperitoneal route as previously described;^15^ furthermore, the intravenous route was associated with improved recovery of clinical indicators of hematological and vascular health.

## MATERIALS AND METHODS

*Mice* - The Institute of Science and Technology in Biomodels (ICTB) of FIOCRUZ provided pathogen-free, female, 8-12-week-old C57BL/6 mice weighing 16-20 g. A 12-hour light/dark cycle and a constant temperature of 21-22ºC were maintained. Animals had free access to water and chow.

*Infection with Plasmodium berghei ANKA (PbA)* - PbA (Malaria Research and Reference Reagent Resource Center, Manassas, VA, USA) were removed from liquid nitrogen and brought to room temperature before injection into C57BL/6 mice via the intraperitoneal (ip) route. Experimental groups of mice were subsequently inoculated with 1×10^6^ infected RBC from the passage mice (day 0). Parasitemia was assessed on day 6 by flow cytometry or Giemsa-stained blood smears. The onset of cerebral malaria in mice was assessed by neurological signs and rectal temperature, using a thermocouple probe (Oakton^®^ Acorn TM; Oakton Instruments, IL, USA).

*Treatments* - In one set of experiments, symptomatic hypothermic mice (31-36ºC)^25^ were randomly divided into three equal groups on day 6 post inoculation to compare the effect of different treatments on survival: (i) artemether only; (ii) artemether plus 200 μL of blood; and (iii) artemether plus 200 μL of plasma. Blood and plasma were obtained from healthy C57BL/6 mice (detailed below). The volume transfused (200 μL, approximately equivalent to 10 mL/kg) was previously defined.^15^ Artemether 20 mg/mL (Dafra Pharma GmbH, Basel, Switzerland) was injected intraperitoneally at 20 mg/kg.^25^ Healthy uninfected mice as well as untreated mice with ECM were studied for comparison. Because artemether plus plasma showed worse survival than artemether alone, this group was omitted from subsequent experiments (see Results).

*Intravenous blood transfusion, drug treatment, and sample collection* - Whole blood was collected from healthy adult anesthetized C57BL/6 mice by cardiac puncture using a heparinized syringe. Whole blood was pooled before being used for transfusion. To obtain plasma, whole blood was separated via centrifugation at 6,000 rpm for 6 minutes. Blood collection and transfusion occurred on day 6 post inoculation with PbA.

All mice with ECM assigned to antimalarial drug treatment received a single intraperitoneal injection of artemether (20 mg/kg), and then were randomly divided into two groups; one group received whole blood via the right jugular vein and the other group underwent a sham transfusion procedure. Briefly, after being anesthetized with 75-100 mg/kg ketamine and 10 mg/kg xylazine, mice were kept in a supine position. Jugular vein was exposed by making a small incision on the neck. A 30-G needle on an insulin syringe was passed through the pectoral muscle and into the lumen of the jugular vein. 200 μL of pooled whole blood or plasma was slowly administered by hand. A sham procedure, involving all steps except the infusion of blood, was carried out on mice assigned to receive artemether alone. After transfusion, the incision was sutured with braided silk, and the animal was returned to the cage and observed until it recovered from anesthesia. The intravenous injection procedure using the jugular vein has been used before in mice to inject smaller volumes (50 μL) of bone marrow mononuclear cell suspensions.^26^ ^,^ ^27^

Sample collection included collection of whole blood and plasma immediately prior to transfusion and at 6 h and 24 h post transfusion. Samples were collected at the same time points for sham-transfused animals. For hematological analysis, whole blood was collected in EDTA-coated microtubes through cardiac puncture. For biochemical analysis, plasma was aspirated from whole blood after separation via centrifugation at 6,000 rpm for 6 minutes, distributed in small aliquots, and stored at −80ºC. The same procedure was performed on uninfected mice and untreated ECM mice which served as comparators.

*Survival experiments* - Survival experiments were performed to compare artemether alone versus artemether plus intravenous plasma versus artemether plus intravenous whole blood. ECM mice (day 6 of infection, rectal temperature of 31-36ºC) were randomly assigned to three treatments groups: (1) artemether 20 mg/kg ip, (2) artemether 20 mg/kg ip plus 200 μL of whole blood iv and (3) artemether 20 mg/kg ip plus 200 μL of plasma iv. Artemether 20 mg/kg artemether ip was administered daily for four more consecutive days. After the last dose of artemether, mice were followed for seven more days and then euthanized with pentobarbital.

*Humane endpoints* - On day 6 of infection by PbA, mice were clinically checked early in the morning for signs of ECM and rectal temperature was measured. Mice that fell within the objective criterion defining ECM (rectal temperature between 31-36ºC) were selected for the experiments. In the eventual circumstance that infected mice showed temperature below 31ºC at this moment, they were immediately subjected to euthanasia. In order to reduce suffering, animals were euthanized via i.p. injection of sodium pentobarbital (200 to 250 mg/kg) followed by cervical dislocation. In case mice showed temperature above 36ºC at this moment, they were checked again in the afternoon and either included in the experimental group (treatment) or subjected to euthanasia. Once treated, mice were followed up every 3-6 h and were subjected to euthanasia if the rectal temperature dropped below 31ºC or if they developed coma, convulsions, or difficulty in breathing. Survival experiments lasted from six to 18 days after infection. Infected mice used as ECM controls were subjected to euthanasia at day 6 and mice with ECM that were treated were followed for another 6 or 24 h. For any invasive intervention prior to euthanasia, such as cardiac puncture for blood collection of Evans blue injection, mice were anesthetized with 75-100 mg/kg ketamine and 10 mg/kg xylazine. All personnel handling the animals received appropriate training.

*Study of blood hematological components* - At time 0 h, 6 h and 24 h, whole blood samples were collected from control and treated animals and sent to ICTB of FIOCRUZ for hematological components analysis, using a pocH-100i automated hematology analyzer (Sysmex).

*Enzyme-linked immunosorbent assays (ELISA) for biochemical analysis of plasma components* - To determine levels of Angiopoietin 1 & 2, Mouse Angiopoietin 1 & 2 Picokine ELISA kits (Boster) were used. Prestored plasma samples were brought to room temperature and subjected to 1:5 dilution in phosphate-buffered saline (PBS) and the ELISA performed according to the manufacturer’s instructions. For haptoglobin measurement, plasma samples were diluted 1:5,000 and analyzed by ELISA (Douset).

*Assessment of blood-brain barrier permeability* - To assess the permeability of the blood-brain barrier at 6 h and 24 h after treatment, the animals were anesthetized with urethane (2 mg/g ip). Each animal received an intravenous injection via the retroorbital plexus of 150 µL of 2% Evans blue stain (Sigma) diluted in 1X PBS. One hour after dye injection, the animals were euthanized, perfused transcaridally with 10 mL of ice-cold saline, and the brains removed. Healthy controls and ECM untreated mice went through same procedure. Brains were incubated in 3 mL of 99.5% formamide (Sigma) for 48 h at 37ºC to extract the dye. One hundred μL of the formamide solution from each brain specimen was evaluated by absorbance spectrophotometry at a wavelength of 630 nm. Values were determined based on a standard curve.

*Ethics* - All experimental work involving animals was ratified by Animal Welfare Committee of the FIOCRUZ (CEUA/FIOCRUZ), under license number L-037/21.

## RESULTS

**Fig. 1:**
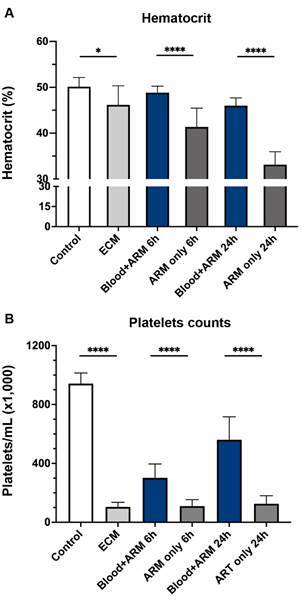
effect of adjuvant intravenous blood transfusion at 6 and 24 hours on levels of hematocrit and platelet counts in mice with experimental cerebral malaria (ECM). (A) Hematocrit: In ECM mice, pretreatment and pretransfusion hematocrit was 8% less than in healthy controls (mean; 46.16% versus 50.12%, p = 0.0390). Mice treated with artemether (ARM) alone showed further decrease in hematocrit after 6 and 24 h (p < 0.0001). Intravenous blood transfusion plus ARM stabilized hematocrit levels at 6 and 24 h (mean 48.8% and 46%, respectively). (B) Platelet count: ECM mice displayed around 90% reduction in platelet counts in relation to healthy controls (p < 0.0001). Intravenous blood transfusion led to partial recovery in platelet counts at 6 h (2.9-fold; p < 0.0001) and at 24 h (5.4-fold; p < 0.0001) compared to mice that received artemether alone. Data are shown as mean ± standard deviation. For comparing groups at a given timepoint, Student’s t-test was performed.

*Intravenous blood transfusion prevents artemether-induced anemia* - To mimic a clinically relevant scenario in which children must present to medical attention with neurological signs before treatment can be initiated, treatment of ECM mice with artemether alone (20 mg/kg ip), artemether plus plasma (200 µL iv), or artemether plus whole blood (200 µL iv) was given after neurological symptoms developed on day 6 post inoculation. Mice with ECM had lower hematocrit than healthy mice on day 6 (46.2 ± 4.18% vs 50.1 ± 2.02%; p = 0.0297) prior to artemether treatment (Fig. 1A). After artemether treatment alone, hematocrit declined rapidly to 41.4 ± 4.10% at 6 h post-treatment and 33.1 ± 2.80% at 24 h post-treatment. In contrast, mice with ECM who were treated with artemether plus whole blood iv maintained hematocrits of 48.6 ± 1.39% at 6 h (p = 0.0004) and 46.0 ± 1.71% at 24 h (p < 0.0001, compared to mice receiving only artemether).

*Intravenous blood transfusion improves ECM-induced thrombocytopenia* - Mice with ECM developed severe thrombocytopenia on day 6 post infection (104,000 ± 31,470 platelets per µL) compared to healthy mice (942,000 ± 72,190 per µL; p < 0.0001), a fall of 90% (Fig. 1B). Treatment with artemether alone had little effect on platelet counts at 6 h (110,000 ± 44,980 per µL) and 24 h (125,700 ± 54,450 per µL). Intravenous blood transfusion as an adjuvant to artemether significantly hastened the recovery of platelets at 6 h (2.7-fold - 301,400 ± 94,880 per µL; p < 0.0001) and at 24 h (4.4-fold - 560,200 ± 156,700 per µL; p < 0.0001) compared to ECM mice treated with artemether only. Intravenous blood transfusion recovered the platelet count in 24 h to around 60% of that noticed in healthy controls, whereas artemether alone had no affect (13% of controls).

**Fig. 2:**
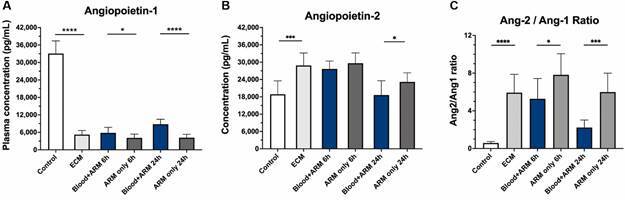
effect of intravenous whole blood transfusion plus artemether on plasma angiopoietin-1 (Ang-1) and angiopoietin-2 (Ang-2) profiles in mice with experimental cerebral malaria (ECM). Plasma levels of angiopoietins among studied groups (healthy controls, blood plus artemether “ARM” and artemether alone) were measured using enzyme-linked immunosorbent assays (ELISA) (n = 10 mice per group). Mice with ECM showed depleted plasma Ang-1 levels (A) compared to uninfected controls (p < 0.0001). Treatment of ECM mice with artemether had no effect on Ang-1 levels, which remained very low. However, Ang-1 levels were slightly (mean 43%) but significantly higher in ECM mice that received whole blood transfusion compared to mice that received artemether alone at 6 h (p = 0.0499) and at 24 h whole blood transfusion led to a 2-fold increase in Ang-1 levels compared to artemether alone (p < 0.0001). (B) Plasma Ang-2 levels increased by 1.5 fold in ECM mice compared to normal controls (p = 0.0001). Treatment with artemether alone or artemether plus whole blood transfusion did not modify the increased Ang-2 levels 6 h after treatment. At 24 h, ECM mice that received whole blood transfusion showed plasma Ang-2 levels back to normal, whereas ECM mice that received artemether alone still showed increased levels (p = 0.0462). (C) The ratio of Ang-2 to Ang-1 was increased 10-fold in ECM mice compared to healthy controls (p < 0.0001). The levels kept increasing after 6 h of artemether alone treatment but stabilized with artemether plus whole blood treatment (p = 0.0357). Within 24 h, Ang-2 levels were still high in ECM mice that received artemether only, but were much lower in ECM mice that received artemether plus whole blood transfusion (p = 0.0002). Data are shown as mean ± standard deviation. For comparing groups at a given timepoint, Student’s t-test was performed.

*Blood transfusion improves endothelial quiescence by restoring the balance of angiopoietin-1 and 2* - Plasma levels of Ang-1 and Ang-2 were assessed before and after treatment with artemether or artemether plus whole blood iv. On day 6 post inoculation, mice with ECM had lower Ang-1 (5,218 ± 1,443 pg/mL), higher Ang-2 (28,842 ± 4,321 pg/mL), and higher Ang-2/Ang-1 ratios (5.93 ± 1.95) than healthy mice (Ang-1: 33,070 ± 4,364 pg/mL, p < 0.0001; Ang-2: 18,893 ± 4,635 pg/mL, p = 0.0006; Ang-2/Ang1: 0.58 ± 0.16, p = 0.0002) (Fig. 2A-C). Following treatment with artemether alone, Ang-1 remained low at 6 (4,092 ± 1,298 pg/mL) and 24 (4,169 ± 1,178 pg/mL) h, Ang-2 remained elevated at 6 (29,628 ± 3,578 pg/mL) and 24 (23,157 ± 3,162 pg/mL) h, and the ratio of Ang-2/Ang-1 remained elevated at 6 (7.82 ± 2.23) and 24 (5.99 ± 2.01) h. In contrast, mice treated with artemether plus whole blood iv showed improvements in angiopoietin balance with higher Ang-1 at 6 (5,837 ± 1,879 pg/mL, p = 0.0485) and 24 (8,781 ± 1,714 pg/mL, p < 0.0001) h, lower Ang-2 at 24 h (18,606 ± 4,963 pg/mL; p = 0.0462), and lower Ang-2/Ang-1 ratios at 6 (5.27 ± 2.15; p = 0.0357) and 24 (2.22 ± 0.80; p = 0.0002) h compared to artemether alone-treated mice. Together, these data indicate that intravenous whole blood transfusion helps to restore angiopoietin balance in artemether-treated mice with ECM.

**Fig. 3:**
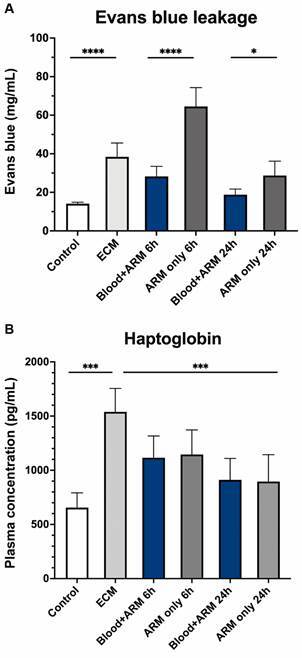
blood-brain barrier (BBB) permeability and plasma levels of haptoglobin in mice with experimental cerebral malaria (ECM) after treatment with artemether with or without intravenous blood transfusion. (A) The BBB permeability (Evans blue assay) in mice with ECM was increased 2.7-fold in relation to healthy controls (p < 0.0001). BBB permeability kept increasing (to 4.6-fold) 6 h after treatment with artemether (ARM) alone, but intravenous transfusion of whole blood prevented the further increase. Indeed, Evans blue leakage in the brain of transfused mice was less than half that observed in mice receiving artemether alone (p < 0.0001). At 24 h, BBB leakage decreased but was still evident in mice treated with artemether alone, whereas it returned to normal levels in mice that received artemether plus blood transfusion (p = 0.0126). (B) Haptoglobin levels in whole plasma was assessed using enzyme-linked immunosorbent assays (ELISA). Mice with ECM showed a mean 135% increase in plasma haptoglobin levels compared to uninfected controls (p < 0.0001). The levels of haptoglobin showed a decline in both treatment groups at 6 and 24 h. No difference between the treatments was noticed any time point. Data are shown as mean ± standard deviation. For comparing groups at a given timepoint, Student’s t-test was performed.

*Intravenous blood transfusion prevents blood-brain barrier (BBB) breakdown* - To investigate whether the improved angiopoietin balance was accompanied by preservation of the blood barrier, Evans blue dye extravasation was measured in mice with ECM treated with artemether alone compared to artemether plus iv blood transfusion. Mice with ECM showed increased Evans blue dye extravasation (38.41 ± 7.15 mg/mL) into the brain parenchyma compared to healthy mice (14.09 ± 0.78 mg/mL; p < 0.0001), an indication that the blood brain barrier was disrupted. Mice with ECM treated with artemether alone exhibited increased disruption of the blood brain barrier 6 h after treatment (64.52 ± 9.80 mg/mL), which partially recovered at 24 h (28.71 ± 7.46 mg/mL). In contrast, mice with ECM treated with artemether plus iv blood transfusion showed improvement of blood brain barrier integrity compared to artemether-only treated mice at 6 (28.25 ± 5.22 mg/mL; p < 0.0001) and 24 (18.81 ± 2.88 mg/mL; p = 0.0126) h post-treatment (Fig. 3A).

*Plasma haptoglobin levels after intravenous blood transfusion* - Haptoglobin was higher in mice with ECM compared to healthy mice (1,539 ± 217 pg/mL vs 655 ± 136 pg/mL; p = 0.0006; Fig. 3B). Treatment with artemether led to lower haptoglobin levels at 6 h (1,116 ± 201 pg/mL) and 24 h (911 ± 198 pg/mL) after treatment, which were similar to mice treated with artemether plus iv blood transfusion at 6 h (1,145 ± 227 pg/mL) and at 24 h (896 ± 248 pg/mL).

**Fig. 4:**
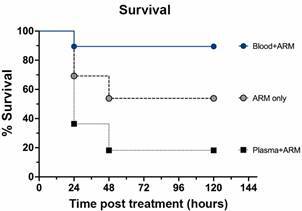
improved survival after blood transfusion plus artemether compared to artemether alone or plasma plus artemether in mice with experimental cerebral malaria (ECM). *Plasmodium-berghei* ANKA-infected mice showing signs of ECM and rectal temperatures between 31 and 36ºC on day 6 of infection were treated with either: (i) artemether 20 mg/kg given intraperitoneally (ip) alone (n = 13); (ii) artemether ip plus 200 μL of whole blood given intravenously (iv) (n = 19) or; (iii) artemether ip plus 200 μL of plasma iv (n = 11). Whole blood or plasma were given only once, together with first artemether dose, whereas artemether was given daily for a total of five days. Adjunctive therapy with whole blood resulted in marked improved survival (90%) compared to artemether alone (54%; p = 0.0004). On the other hand, plasma therapy with artemether resulted in worsened outcome, with only 18% survival. Three separate experiments were performed, and the results were combined. For statistical analysis, log-rank Mantell-Cox test was performed.

*Artemether plus intravenous whole blood transfusion improves survival of mice with ECM compared to artemether plus plasma or artemether alone* - PbA-infected mice that present with signs of ECM typically die within 24 h if not treated. Mice with ECM that received artemether had a survival rate of 7/13 (54%) (Fig. 4) and survival increased to 17/19 (90%) when artemether was combined with iv whole blood transfusion. In contrast, the combination of artemether plus iv plasma transfusion lowered survival to 2/18 (18%).

Overall, iv whole blood transfusion improved anemia, thrombocytopenia, angiopoietin balance, blood brain barrier integrity, and survival of artemether-treated mice with ECM.

## DISCUSSION

Clinical observational studies have suggested that whole blood transfusion may improve survival of children with cerebral malaria even in the absence of severe anemia. However, the mechanisms underlying the apparent protection of whole blood remain undefined. Here, we find that in a murine model of cerebral malaria, intravenous whole blood restored the balance of angiopoietin 1 and 2, key factors that regulate endothelial inflammation and barrier integrity. Indeed, intravenous whole blood transfusion prevented the blood brain barrier breakdown that is a cardinal feature of experimental cerebral malaria and improved survival. Notably, infusion of plasma instead of whole blood did not convey any protection and instead worsened survival. Thus, we must infer that red blood cells are a necessary component of the endothelial-preserving property of whole blood.

Prior work has demonstrated the therapeutic efficacy of whole blood transfusion in PbA-infected C57BL/6 mice with ECM, using intraperitoneal route for blood administration, resulting in 76% survival compared to 51% survival in ECM mice receiving artemether only.^15^ In the previous study, the intraperitoneal route of transfusion was chosen as a proof of concept due to the difficulties of performing intravenous injection of large volumes in mice with ECM. However, clinically, blood transfusion is normally given via the intravenous route, and thus a solution was achieved by means of intrajugular injection. The present study demonstrates that a single intrajugular transfusion of whole blood along with artemether resulted in 90% survival of mice with ECM. This strategy had stronger effects on hematocrit, platelet counts, Ang-1 and Ang-2 levels and blood-brain barrier integrity compared to intraperitoneal transfusion.

There may be several reasons for the better performance of intravenous over intraperitoneal transfusion. Intravenous injection makes fresh red blood cells and other components (plasma, platelets, etc.) immediately available intravascularly, therefore any benefits in terms of vascular health, perfusion and oxygen carrying capacity start to take place right away. In the case of intraperitoneal transfusion, the dynamics of transfer of the transfused components to the vascular system is not clear, but there may be a time gap between injection and the full availability of the components in the circulation. In addition, not all red blood cells transfused by intraperitoneal route actually reach the circulation,^24^ and the same may be true for plasma. Therefore, the impact of intravenous transfusion, which is rapid and without losses, was anticipated to be more efficacious.

The beneficial effects of blood transfusion include improvement in red blood cell deformability^28^ ^,^ ^29^ and oxygen-carrying capacity of the blood, which in the setting of cerebral ischemia may be a critical advantage for the patient. Lower hematocrit also results in decreased vascular wall shear stress,^30^ with decreased eNOS activity, which together with the nitric oxide-scavenging action of free hemoglobin leads to worsened endothelial dysfunction. Under conditions of decreased oxygen saturation, fresh red blood cells may induce vasodilation by exporting NO bioactivity,^31^ release of ATP from RBCs also results in RBC-mediated hypoxic vasodilation.^32^ ^,^ ^33^ Therefore, increasing hematocrit through whole blood transfusion should help restore endothelial vasoregulatory function and improve tissue perfusion.

Endothelial cell activation and dysfunction are hallmarks of malaria pathogenesis.^34^ ^,^ ^35^ ^,^ ^36^ Endothelial protein markers such as angiopoietins have been found to play a critical physiological role in maintenance of vascular integrity. It has been previously shown that the levels of the endothelial cell quiescence promoter Ang-1 are depleted, whereas the levels of endothelial cell activation/inflammation promoter Ang-2 are elevated, in mice with ECM.^15^ It is noteworthy that blood transfusion given intraperitoneally had no effect on restoring Ang-1 levels, but when given intravenously an increase in Ang-1 levels was observed after 6 h, and a more robust increase was seen at 24 h. And while Ang-2 levels were equally restored by either intraperitoneal or intravascular transfusion, intravenous transfusion had a stronger effect on Ang-1 levels resulting in an improved Ang-2/Ang-1 ratio. Since Ang-1 and Ang-2 compete for binding to Tie2, with opposite effects, this improved profile achieved by intravascular transfusion likely results in a faster recovery of endothelial function. Indeed, reversing Ang-2/1 imbalance promotes endothelial cell survival, stabilizes endothelial interactions with supporting cells and limits vascular permeability.^37^ ^,^ ^38^ ^,^ ^39^ Ang-1 stabilizes the blood brain barrier while Ang-2 weakens the pericyte-endothelial cell interaction, resulting in blood brain barrier disruption.^36^ ^,^ ^37^ ^,^ ^38^ ^,^ ^39^ ^,^ ^40^ Since severity of cerebral malaria is related to vascular dysfunction^23^ and much of the mortality occurs in the first 24 h after antimalarial treatment, this faster recovery of endothelial function may be a critical factor explaining the substantial increase in survival among transfused animals.

A faster recovery of endothelial function may help to explain a more rapid reversal of blood brain barrier breakdown. The overall profile of Evans blue leakage observed after treatment of ECM mice with artemether or artemether plus intravenous blood transfusion was similar to that observed in the previous study using intraperitoneal transfusion.^15^ However, ECM mice receiving intravenous transfusion showed a more profound recovery of blood brain barrier integrity, both at 6 and 24 h after treatment. These findings are in line with a better profile of plasma Ang-1 and Ang-2/Ang-1 levels and with a better survival outcome. In the case of haptoglobin levels, the effects of intravenous transfusion were not different from those observed with intraperitoneal transfusion.^15^

Malaria is a true hematological infectious disease, heavy parasite burden and hemolysis of parasitized and non-parasitized red blood cells may result in rapid decline of hematocrit.^41^ ^,^ ^42^ Intravenous transfusion again resulted in a better hematological outcome compared to intraperitoneal transfusion. Whereas the latter only partially prevented the marked fall of hematocrit following artemether treatment observed in non-transfused mice,^15^ intravenous transfusion resulted in a more consistent maintenance of hematocrit. This can be explained in part by the fact that not all red blood cells transfused by intraperitoneal route actually reach the circulation.^24^ But there is also indication that blood transfusion does not lead only to a passive recovery of circulating red blood cell numbers. The magnitude of hematocrit recovery at 24 h in transfused compared to non-transfused mice was greater than expected given the amount of red blood cells transfused. Therefore, whole blood transfusion may either induce an active response of the recipient mice, enhancing bone marrow production of new red blood cells, or transfusion has a protective effect on the hemolytic effect of artemether. While the specific mechanisms responsible for the improved hematological indices remain to be elucidated, higher hematocrit has beneficial effects such as increasing shear stress,^30^ managing acid-base balance,^13^ ^,^ ^43^ improving red blood cell deformability, and reducing microvascular obstruction - all of which improve brain tissue oxygen delivery.^44^ ^,^ ^45^ ^,^ ^46^

Another one of the more well-known hematologic changes observed in patients with malaria is thrombocytopenia.^47^ *P. falciparum* infected patients with a platelet count below 20 × 10^9^/L were five times more likely to die than patients with a higher platelet count.^48^ Intraperitoneal blood transfusion partially restored platelet counts in ECM mice.^15^ Here again, the effect of intravenous blood transfusion was stronger, leading to a faster recovery of platelet counts. The magnitude of the recovery was greater than expected by a passive transfer of platelets contained in the transfused blood. Because platelets have been implicated with the pathogenesis of ECM, in processes such as coagulopathy,^49^ endothelial activation,^34^ cytoadherence^50^ and auto-agglutination,^51^ restoring fresh platelets in a timely manner may be helpful in preventing mortality.

Finally, this study confirmed that, while whole blood transfusion is an intervention with high efficacy in preventing death by ECM, healthy plasma transfusion has the opposite effect, leading to increased mortality. This is probably related to increased circulating volume without increased oxygen carrying capacity which may worsen cardiovascular performance and potentially exacerbate brain swelling and pulmonary edema.^52^ ^,^ ^53^

Collectively our data suggest that intravenous blood transfusion reversed anemia and thrombocytopenia, facilitated restoration of endothelial quiescence, and improved vascular integrity leading to improved survival in artemether-treated experimental CM. These findings deserve further scrutiny to better understand the mechanisms behind the benefit achieved and to provide additional support for translation of these findings to human patients.

## References

[1] WHO (2020). World malaria report 2020. 20 years of global progress and challenges.

[2] Boivin MJ, Bangirana P, Byarugaba J, Opoka RO, Idro R, Jurek AM (2007). Cognitive impairment after cerebral malaria in children: a prospective study. Pediatrics.

[3] John CC, Bangirana P, Byarugaba J, Opoka RO, Idro R, Jurek AM (2008). Cerebral malaria in children is associated with long-term cognitive impairment. Pediatrics.

[4] Marsh K, Forster D, Waruiru C, Mwangi I, Winstanley M, Marsh V (1995). Indicators of life-threatening malaria in African children. N Engl J Med.

[5] Dondorp AM, Fanello CI, Hendriksen IC, Gomes E, Seni A, Chhaganlal KD (2010). Artesunate versus quinine in the treatment of severe falciparum malaria in African children (AQUAMAT) an open-label, randomised trial. Lancet.

[6] Cooke BM, Stuart J, Nash GB (2014). The cellular and molecular rheology of malaria. Biorheology.

[7] Wyler DJ, Quinn TC, Chen LT, Wyler DJ, Quinn TC, Chen L-T (1981). Relationship of alterations in splenic clearance function and microcirculation to host defense in acute rodent malaria. J Clin Invest.

[8] Wassmer SC, Grau GER (2016). Platelets as pathogenetic effectors and killer cells in cerebral malaria. Expert Rev Hematol.

[9] Conroy AL, Lafferty EI, Lovegrove FE, Krudsood S, Tangpukdee N, Liles WC (2009). Whole blood angiopoietin-1 and -2 levels discriminate cerebral and severe (non-cerebral) malaria from uncomplicated malaria. Malar J.

[10] Elphinstone RE, Riley F, Lin T, Higgins S, Dhabangi A, Musoke C (2015). Dysregulation of the haem-haemopexin axis is associated with severe malaria in a case-control study of Ugandan children. Malar J.

[11] Gramaglia I, Sobolewski P, Meays D, Contreras R, Nolan JP, Frangos JA (2006). Low nitric oxide bioavailability contributes to the genesis of experimental cerebral malaria. Nat Med.

[12] Mandala WL, Msefula CL, Gondwe EN, Drayson MT, Molyneux ME, MacLennan CA (2017). Cytokine profiles in Malawian children presenting with uncomplicated malaria, severe malarial anemia, and cerebral malaria. Clin Vaccine Immunol.

[13] Ackerman H, Ayestaran A, Olola CHO, Jallow M, Agbenyega T, Bojang K (2020). The effect of blood transfusion on outcomes among African children admitted to hospital with Plasmodium falciparum malaria a prospective, multicentre observational study. Lancet Haematol.

[14] Ackerman H, Olola CHO, Krishna S, Roberts DJ, Kremsner PG, Newton CR (2021). Time-to-death is a potential confounder in observational studies of blood transfusion in severe malaria - Authors' reply. Lancet Haematol.

[15] Gul S, Ribeiro-Gomes FL, Moreira AS, Sanches GS, Conceição FG, Daniel-Ribeiro CT (2021). Whole blood transfusion improves vascular integrity and increases survival in artemether-treated experimental cerebral malaria. Sci Rep.

[16] Cabrales P, Zanini GM, Meays D, Frangos JA, Carvalho LJM (2011). Nitric oxide protection against murine cerebral malaria is associated with improved cerebral microcirculatory physiology. J Infect Dis.

[17] Zanini GM, Cabrales P, Barkho W, Frangos JA, Carvalho LJM (2011). Exogenous nitric oxide decreases brain vascular inflammation, leakage and venular resistance during Plasmodium berghei ANKA infection in mice. J Neuroinflammation.

[18] Ong PK, Moreira AS, Daniel-Ribeiro CT, Frangos JA, Carvalho LJM (2018). Reversal of cerebrovascular constriction in experimental cerebral malaria by L-arginine. Sci Rep.

[19] Moreira AS, Estato V, Malvar DC, Sanches GS, Gomes F, Tibirica E (2019). L-arginine supplementation, and thromboxane synthase inhibition increases cerebral blood flow in experimental cerebral malaria. Sci Rep.

[20] Carvalho LJM, Lenzi HL, Pelajo-Machado M, Oliveira DN, Daniel-Ribeiro CT, Ferreira-da-Cruz MF (2000). Plasmodium berghei cerebral Malaria in CBA mice is not clearly related to plasma TNF levels or intensity of histopathological changes. Exp Parasitol.

[21] Zanini GM, Cabrales P, Barkho W, Frangos JA, Carvalho LJM (2011). Exogenous nitric oxide decreases brain vascular inflammation, leakage and venular resistance during Plasmodium berghei ANKA infection in mice. J Neuroinflammation.

[22] Cabrales P, Zanini GM, Meays D, Frangos JA, Carvalho LJM (2010). Murine cerebral malaria is associated with a vasospasm-like microcirculatory dysfunction, and survival upon rescue treatment is markedly increased by nimodipine. Am J Pathol.

[23] Carvalho LJM, Moreira AS, Daniel-Ribeiro CT, Martins YC (2014). Vascular dysfunction as a target for adjuvant therapy in cerebral malaria. Mem Inst Oswaldo Cruz.

[24] Al Shoyaib A, Archie SR, Karamyan VT (2020). Intraperitoneal route of drug administration should it be used in experimental animal studies?. Pharm Res.

[25] Clemmer L, Martins YC, Zanini GM, Frangos JA, Carvalho LJM (2011). Artemether and artesunate show the highest efficacies in rescuing mice with late-stage cerebral malaria and rapidly decrease leukocyte accumulation in the brain. Antimicrob Agents Chemother.

[26] Maron-Gutierrez T, Silva JD, Asensi KD, Bakker-Abreu I, Shan Y, Diaz BL (2013). Effects of mesenchymal stem cell therapy on the time course of pulmonary remodeling depend on the etiology of lung injury in mice. Crit Care Med.

[27] Lima MN, Oliveira HA, Fagundes PM, Estato V, Silva AYO, Freitas RJRX (2020). Mesenchymal stromal cells protect against vascular damage and depression-like behavior in mice surviving cerebral malaria. Stem Cell Res Ther.

[28] Meremikwu MM, Smith HJ (2000). Blood transfusion for treating malarial anaemia. Cochrane Database Syst Rev.

[29] Dondorp AM, Nyanoti M, Kager PA, Mithwani S, Vreeken J, Marsh K (2002). The role of reduced red cell deformability in the pathogenesis of severe falciparum malaria and its restoration by blood transfusion. Trans R Soc Trop Med Hyg.

[30] Cabrales P, Intaglietta M, Tsai AG (2007). Transfusion restores blood viscosity and reinstates microvascular conditions from hemorrhagic shock independent of oxygen carrying capacity. Resuscitation.

[31] Powloski JR, Hess DT, Stamler JS (2001). Export by red blood cells of nitric oxide bioactivity. Nature.

[32] Chinar E, Zhou S, DeCourcey J, Wang Y, Waugh RE, Wan J (2015). Piezo1 regulates mechanotransductive release of ATP from human RBCs. Proc Natl Acad Sci USA.

[33] Ellsworth ML, Ellis CG, Sprague RS (2016). Role of erythrocyte-released ATP in the regulation of microvascular oxygen supply in skeletal muscle. Acta Physiol (Oxf).

[34] Hollestelle MJ, Donkor C, Mantey EA, Chakravorty SJ, Craig A, Akoto AO, et al von Willebrand factor propeptide in malaria: evidence of acute endothelial cell activation (2006). Br. J Haematol.

[35] Yeo TW, Lampah DA, Gitawati R, Tjitra E, Kenangalem E, Piera K (2008). Angiopoietin-2 is associated with decreased endothelial nitric oxide and poor clinical outcome in severe falciparum malaria. Proc Natl Acad Sci USA.

[36] Lovegrove FE, Tangpukdee N, Opoka RO, Lafferty EI, Rajwans N, Hawkes M (2009). Serum angiopoietin-1 and -2 levels discriminate cerebral malaria from uncomplicated malaria and predict clinical outcome in African children. PLoS One.

[37] Papapetropoulos A, García-Cardeña G, Dengler TJ, Maisonpierre PC, Yancopoulos GD, Sessa WC (1999). Direct actions of angiopoietin-1 on human endothelium evidence for network stabilization, cell survival, and interaction with other angiogenic growth factors. Lab Investig.

[38] Jeansson M, Gawlik A, Anderson G, Li C, Kerjaschki D, Henkelman M (2011). Angiopoietin-1 is essential in mouse vasculature during development and in response to injury. J Clin Invest.

[39] Gavard J, Patel V, Gutkind JS (2008). Angiopoietin-1 prevents VEGF-induced endothelial permeability by sequestering Src through mDia. Dev Cell.

[40] Conroy AL, Phiri H, Hawkes M, Glover S, Mallewa M, Seydel KB (2010). Endothelium-based biomarkers are associated with cerebral malaria in Malawian children a retrospective case-control study. PLoS One.

[41] Wang T, Xing Z (2018). Local hematocrit fluctuation induced by malaria-infected red blood cells and its effect on microflow. Biomed Res Int.

[42] La'lang M.Budhy TI.Prastyawati R (2021). Description of hematocrit in Malaria tropica (Plasmodium falciparum) patients at Jayapura Regional General Hospital. Malaysian J Med Heal Sci.

[43] Leisewitz AL, Guthrie AJ, Berry WL (1996). Evaluation of the effect of whole-blood transfusion on the oxygen status and acid-base balance of Babesia canis infected dogs using the oxygen status algorithm. J S Afr Vet Assoc.

[44] Hanson J, Lam SWK, Mahanta KC, Pattnaik R, Alam S, Mohanty S (2012). Relative contributions of macrovascular and microvascular dysfunction to disease severity in falciparum malaria. J Infect Dis.

[45] Ishioka H, Ghose A, Charunwatthana P, Maude R, Plewes K, Kingston H (2015). Sequestration and red cell deformability as determinants of hyperlactatemia in falciparum malaria. J Infect Dis.

[46] Salmen M, Hendriksen S, Gorlin J, Leclaire M, Prekker ME (2017). Oxygen delivery during severe anemia when blood transfusion is refused on religious grounds. Ann Am Thorac Soc.

[47] Ladhani S, Lowe B, Cole AO, Kowuondo K, Newton CRJC (2002). Changes in white blood cells and platelets in children with falciparum malaria relationship to disease outcome. Br J Haematol.

[48] Hanson J, Phu NH, Hasan MU, Charunwatthana P, Plewes K, Maude RJ (2015). The clinical implications of thrombocytopenia in adults with severe falciparum malaria a retrospective analysis. BMC Med.

[49] Angchaisuksiri P (2014). Coagulopathy in malaria. Thromb Res.

[50] Wassmer SC, Lépolard C, Traoré B, Pouvelle B, Gysin J, Grau GE (2004). Platelets reorient Plasmodium falciparum-infected erythrocyte cytoadhesion to activated endothelial cells. J Infect Dis.

[51] Chotivanich K, Sritabal J, Udomsangpetch R, Newton P, Stepniewska KA, Ruangveerayuth R (2004). Platelet-induced autoagglutination of Plasmodium falciparum-infected red blood cells and disease severity in Thailand. J Infect Dis.

[52] Maitland K, Kiguli S, Opoka RO, Engoru C, Olupot-Olupot P, Akech SO (2011). Mortality after fluid bolus in African children with severe infection. Kenya Med Res Inst (KEMRI)-Well-Come Trust Res Program.

[53] Ackermann H (2013). Management of severe malaria enthusiasm for fluid resuscitation dampened by lung water. Crit Care Med.

